# Genetic recombination shapes complex hybrid effects across the pig genome

**DOI:** 10.1093/nsr/nwag322

**Published:** 2026-05-28

**Authors:** Hai-Bing Xie, Zi-Qin Huang, Li-Gang Wang, Long-Chao Zhang, Shu-Shu Yan, Jia-Kun Deng, Adeniyi C Adeola, Qing-Long Li, Lin Tao, Kui Li, Shu-Hong Zhao, Zhao-Bang Zeng, Li-Xian Wang, Ya-Ping Zhang

**Affiliations:** State Key Laboratory of Genetic Evolution & Animal Models and Yunnan Key Laboratory of Molecular Biology of Domestic Animals, Kunming Institute of Zoology, Chinese Academy of Sciences, Kunming 650223, China; State Key Laboratory of Genetic Evolution & Animal Models and Yunnan Key Laboratory of Molecular Biology of Domestic Animals, Kunming Institute of Zoology, Chinese Academy of Sciences, Kunming 650223, China; Division of Life Sciences and Medicine, University of Science and Technology of China, Hefei 230026, China; Institute of Animal Science, Chinese Academy of Agricultural Sciences, Beijing 100193, China; Institute of Animal Science, Chinese Academy of Agricultural Sciences, Beijing 100193, China; Bio-X Center for Interdisciplinary Innovation, School of Ecology and Environmental Science, Yunnan University, Kunming 650500, China; State Key Laboratory of Genetic Evolution & Animal Models and Yunnan Key Laboratory of Molecular Biology of Domestic Animals, Kunming Institute of Zoology, Chinese Academy of Sciences, Kunming 650223, China; State Key Laboratory of Genetic Evolution & Animal Models and Yunnan Key Laboratory of Molecular Biology of Domestic Animals, Kunming Institute of Zoology, Chinese Academy of Sciences, Kunming 650223, China; Bio-X Center for Interdisciplinary Innovation, School of Ecology and Environmental Science, Yunnan University, Kunming 650500, China; State Key Laboratory of Genetic Evolution & Animal Models and Yunnan Key Laboratory of Molecular Biology of Domestic Animals, Kunming Institute of Zoology, Chinese Academy of Sciences, Kunming 650223, China; Kunming College of Life Science, University of Chinese Academy of Sciences, Kunming 650204, China; University of Chinese Academy of Sciences, Beijing 100049, China; Shenzhen Branch, Guangdong Laboratory of Lingnan Modern Agriculture, Key Laboratory of Livestock and Poultry Multi-omics of MARA, Agricultural Genomics Institute at Shenzhen, Chinese Academy of Agricultural Sciences, Shenzhen 518124, China; Key Laboratory of Agricultural Animal Genetics, Breeding and Reproduction, Ministry of Education & Key Laboratory of Pig Genetics and Breeding, Ministry of Agriculture and Rural Affairs, Huazhong Agricultural University, Wuhan 430070, China; Bioinformatics Research Center, Department of Horticultural Science, North Carolina State University, Raleigh, NC 27607, USA; Institute of Animal Science, Chinese Academy of Agricultural Sciences, Beijing 100193, China; State Key Laboratory of Genetic Evolution & Animal Models and Yunnan Key Laboratory of Molecular Biology of Domestic Animals, Kunming Institute of Zoology, Chinese Academy of Sciences, Kunming 650223, China; Bio-X Center for Interdisciplinary Innovation, School of Ecology and Environmental Science, Yunnan University, Kunming 650500, China

**Keywords:** genetic recombination, hybrid effect, sex difference, pig

## Abstract

Genome and phenotype evolution during hybridization has been a central topic in evolutionary biology, yet the genetic mechanism underlying the hybrid effect variation remain elusive. Here we use the pig as a model and quantify hybrid effects by measuring the phenotypic difference between homozygotes and heterozygotes across 135 traits in 578 F2 individuals from a Eurasian pig cross population. We demonstrate that hybrid effect sizes follow an exponential distribution across the genome. The rate parameter (λ) of this exponential distribution exhibits a hump-shaped pattern with increasing levels of F0 genomic divergence, indicating a genomic transition from inbreeding depression to hybrid vigor and ultimately to hybrid depression. Further analysis reveals that genetic recombination is significantly correlated with the variation in λ, supporting a model in which both recombination and genomic divergence jointly shape the landscape of hybrid effects. Females display lower λ values, with a pronounced reduction in regions exhibiting hybrid depression, revealing a fundamental sex difference in Eurasian pig hybridization. We further show that the λ positively correlates with F2 heterozygote-to-homozygote genotype frequency ratio. This suggests that recombination may introduce advantageous hybrid effect in genetic backgrounds otherwise characterized by inbreeding depression or hybrid depression, resulting in asynchronous evolutionary paces across the genome.

## INTRODUCTION

The role of hybridization remains a contentious issue in evolutionary biology [1]. Numerous explanations have been proposed from diverse perspectives to elucidate its benefits and disadvantages [2–4]. Historically, botanists have argued that hybridization acts as a creative force, driving diversification and adaptive evolution [5], with transgressive segregation identified as important in shaping extreme hybrid phenotypes for environmental adaptation [2,6]. In contrast, zoologists have frequently documented reproductive barriers and reduced hybrid fitness in cases where parental species or populations exhibit moderate to high levels of divergence [7–9]. Irrespective of these distinct evolutionary outcomes, hybridization facilitates the combination of alleles through segregation and recombination during the alternation of meiosis and syngamy in sexual reproduction [10]. This recombination is theorized to mitigate the Hill-Robertson effect and enhance the efficacy of natural selection [11–13]. Given the ubiquity of hybridization and its profound evolutionary implications, the precise role of hybridization and the mechanisms underlying its varied effects remain to be comprehensively elucidated.

Hybrid effects are expected to vary substantially across different hybridization events. These effects have been theorized to range from inbreeding depression and hybrid vigor to hybrid depression in hybridizations as the degree of parental differentiation increases [14,15]. While studies have employed quantitative approaches to assess hybrid effects by comparing hybrid phenotypes with those of parental lines under varying levels of differentiation [16–18], the evolutionary mechanisms underlying the effect variation have rarely been explored. Considering that genomes often evolve at divergent rates and incomplete lineage sorting is common in highly divergent species [19,20], hybrid effects may display significant genomic heterogeneity. In light of this variability, we propose that beneficial hybrid effects could be confined to specific genomic regions, thereby enhancing overall population fitness, while other regions might contribute to relatively detrimental outcomes.

To investigate the hypothesized genomic heterogeneity and explore the genetics underlying the transition of hybrid effects, we adopted a genomic approach to assess the relative probabilities of hybridization introducing beneficial versus detrimental effects across the genome. We selected the pig (*Sus scrofa*) as a model system to study these evolutionary dynamics, as it exhibits both inbreeding depression and hybrid incompatibility across the genome [21–23]. Our experimental design involved analyzing a three-generation family derived from a cross between European Large White (LW) and East Asian Min (MIN) pigs. These two breeds, domesticated independently [24,25], share a common wild boar ancestor dating back approximately one million years [26]. Eurasian pig hybridization has been widely utilized in breeding programs to develop new breeds [27], and the moderate genetic differentiation observed among Eurasian pigs [26,28,29] renders them an ideal system for investigating the genomic distribution of advantageous and deleterious hybrid effects. We conducted the experiment by examining multi-trait hybrid effect variation (the phenotypic difference between homozygotes and heterozygotes) and sex-specific differences alongside the segregation of founder alleles in the F2 generation, and analyzed heterozygote advantage to elucidate genotype–phenotype evolution in Eurasian pig hybridization.

## RESULTS

### Recombination, segregation and exponential distribution of hybrid effect

**Figure 1.**
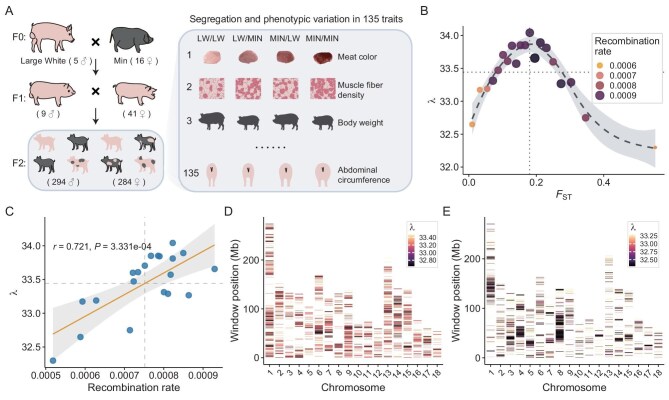
Hybrid effect variation in the LW-MIN F2 population mediated by genetic differentiation and recombination. (A) Schematic illustration of the three-generation LW-MIN pig family and phenotypic variation for hybrid effect analysis. The homozygote-heterozygote phenotypic difference in 135 traits was analyzed to assess the hybrid effect in the LW-MIN F2 population. For each trait, the phenotypic means in 100-kb sliding windows were calculated on individuals sharing the same genotype in each window. The genotypes are denoted as paternal/maternal alleles. (B) Recombination-mediated transition of hybrid effects from inbreeding depression (*F*_ST_ < 0.095 and λ < 33.5) to hybrid vigor (λ > 33.5) and hybrid depression (*F*_ST_ > 0.26 and λ < 33.5), as evidenced by a hump-shaped distribution of λ parameters (averaged between sexes) across twenty bins of 100-kb autosomal windows sorted by genomic differentiation (*F*_ST_). The peak λ of hybrid vigor shows the highest recombination rates. The λ represents the estimated rate parameter of the exponential distribution for hybrid effect sizes across 135 F2 traits, reflecting the probability of beneficial hybrid effects in each bin. Data point sizes scale with the average recombination rates in the bins. The horizontal dotted line indicates the mean of the λ across the 20 window bins, and the vertical dotted line indicates the mean of the *F*_ST_ for all the 100-kb windows. (C) A positive correlation between the λ parameter and recombination rate. (D) Scattered distribution of inbreeding depression effects across the autosomes. (E) Clustered distribution of the hybrid depression effects in long low-recombination regions.

We initiated our analysis on the LW-MIN family by characterizing recombination events during F1 meiosis and allelic segregation in an F2 population of 294 males and 284 females (Fig. 1A), tracing the transmission of genetic material across generations (Fig. S1). Reconstruction of autosomal haplotypes for the entire pedigree revealed a total of 19 551 recombination events (8417 paternal and 11 134 maternal) in the F2 autosomes (Fig. S2). The elevated maternal recombination rate further validates previously reported female-biased distributions [30,31]. Notably, chromosomes 5 (12.0–12.1Mb, *r* = 0.072), 12 (26.4–26.5Mb, *r* = 0.061) and 10 (7.6–7.7Mb, *r* = 0.060) exhibited the highest localized recombination rates (measured as events per transmission in each 100-kb window), whereas chromosomes 1 and 13 displayed extensive recombination deserts (Table S1). This heterogeneity in recombination rates is expected to generate substantial variance in intra-chromosomal linkage disruption, thereby enabling diverse hybrid effects to manifest across autosomes in the F2 population. The genotypes of F2 individuals were determined in 100-kb autosomal sliding windows based on the allele origin of the F0 founders (Fig. S1; see Methods), with MIN/LW and LW/MIN heterozygous genotypes classified as distinct categories.

To comprehensively investigate hybrid effects, we collected and analyzed phenotypic data for 135 traits in the F2 population, spanning diverse physiological domains, including muscle growth, fat deposition, bone size, and organ development (Fig. 1A; Table S2). Hybrid effects were quantified as the phenotypic differences between heterozygous and homozygous genotypes of F2 individuals within each 100-kb window (see Methods). This comparative framework is essential for understanding hybridization, as it elucidates how hybrid effects scale with allelic differentiation between the LW and MIN breeds. The magnitude of these phenotypic differences serves as a proxy for evaluating whether hybridization is likely to confer evolutionary benefits or costs. Our approach aligns with Fisher’s geometric model (FGM) of adaptation [32], which predicts that new mutations with smaller effect sizes are more likely to be advantageous. Extending this framework to hybrid effects by treating homozygotes as wild-type and heterozygotes as mutant genotypes (Fig. S3), smaller phenotypic differences in homozygote-heterozygote comparisons are expected to correlate with a higher probability of beneficial hybrid outcomes.

We analyzed the distribution of hybrid effect sizes across all 135 traits in autosomal windows in each sex. After the normalization of hybrid effects to sex-specific phenotypic means, we found that the effects in both F2 males (the rate parameter λ = 33.61, equal to the reciprocal of the mean effect size) and females (λ = 33.10) followed exponential distributions (Fig. S3). This pattern suggests that exponential scaling is an intrinsic property of hybrid effects, likely driven by temporally overlapping evolutionary processes acting on distinct genomic regions. This finding indicates that hybridization establishes a dynamic equilibrium across the genome, with different genomic regions contributing heterogeneous magnitudes of hybrid effects. The exponential distribution aligns with, yet differs fundamentally from, a theoretical model of adaptive evolution [33,34], in which the fixation sequence of temporally non-overlapping mutations at a locus exhibits a progressive decline in effect sizes at a constant rate as populations approach fitness optima.

### Interplay between genomic differentiation and recombination in shaping hybrid effects

To explore the evolutionary mechanisms driving the hybrid effect variation, we examined the relationship between hybrid effects and genetic differentiation by analyzing how these effects scale with allelic differentiation between the LW and MIN breeds. A genomic comparison between the LW and MIN founder pigs revealed significant genetic differentiation, with a median *F*_ST_ of 0.16 across 100-kb windows (Fig. S4). To obtain a high confidence in estimating λ, we sorted all genomic windows by their *F*_ST_ values and split them into twenty bins with an equal number of windows (except for the first bin after excluding non-positive *F*_ST_) (Table S3). The λ was estimated across the *F*_ST_ bins independently in each sex and here an averaged λ was used to reflect the λ of the total population. Notably, we observed compelling evidence of hybrid effect variation, characterized by a hump-shaped distribution of λ in windows binned by *F*_ST_ (Fig. 1B; Tables S3 and S4). This hump-shaped distribution of λ was further observed in using only continuous traits (Fig. S5) and 1-Mb sliding window (Fig. S5). In fact, an analysis of pooled male and female samples also rendered a similar result with slight difference of the estimated λ (Fig. S5). In the following analysis, we focused only on the analysis of 100-kb windows. Genomic regions displaying a larger λ parameter are indicative of hybrid vigor with a higher probability of being beneficial, whereas those with smaller λ values are associated with inbreeding or hybrid depression. Peak λ values (λ > 33.5) clustered within a moderate differentiation range (0.095 < *F*_ST_ < 0.26, *n* = 12 401 windows), consistent with the evolutionary transition from inbreeding depression (λ < 33.5 and *F*_ST_ < 0.095, *n* = 5136) to hybrid vigor and, ultimately, hybrid depression (λ < 33.5 and *F*_ST_ > 0.26, *n* = 4512) [14–16,35]. Leave-10%-out analysis (with a random selection of 90% traits in 1000 replicates) revealed that hump-shaped distribution across the bins were robust (Fig. S5). Unlike traditional metrics such as better-parent heterosis (BPH) or mid-parent heterosis (MPH), which focus on the scale of phenotypic difference between the hybrids and parental lines [17,18,36,37], the λ captures the probability of beneficial effects [32] and fixation likelihood [38] in the evolutionary contexts (see Materials and Methods).

Notably, the genomic regions within the λ peak—spanning 56.24% of autosomal windows—showed significantly elevated recombination rates (Fig. 1B). We observed a strong positive correlation between λ and recombination rate (Fig. 1C, Pearson correlation test, *r* = 0.72, *P* = 3.33 × 10^−4^), with recombination hotspots tending to cluster specifically in the λ peak. Across hybrid effect categories, average recombination rates were 0.067% in inbreeding depression regions and 0.073% in hybrid depression regions—both lower than the 0.080% average observed in hybrid vigor regions. To test the statistical significance of this modest (0.01%) elevation in recombination rate in hybrid vigor regions, we conducted a permutation test, assuming that windows with varying recombination rates were randomly distributed across the three hybrid effect categories. We found that the elevated recombination rate in hybrid vigor regions is unlikely to be caused by random chance (*P* = 1.28 × 10^−4^; 1.0 × 10^6^ replicates; Fig. S6). Specifically, 60.25% of windows with the highest recombination rates (*r* ≥ 1%) mapped to this region, compared to 59.11% and 55.06% for intermediate (0.01% < *r* < 1%) and low (*r* ≤ 0.01%) recombination rates, respectively. This pattern implies that recombination may act as a driver of hybrid vigor, which is consistent with the role of recombination in reducing the Hill-Robertson effect [12].

The decline in λ on either side of the λ peak reflects either the inbreeding depression due to insufficient genomic differentiation (*F*_ST_ < 0.095) or the hybrid depression resulting from excessive differentiation (*F*_ST_ > 0.26) (Fig. 1B). Notably, the reduction in λ was more pronounced in regions associated with hybrid depression than in those linked to inbreeding depression, suggesting a stronger selective constraint against hybrid depression. Despite the common pattern in λ decline, the genomic distribution of these effects differed, with the inbreeding depression having a scattered distribution across the genome (Fig. 1D), and the hybrid depression exhibiting a clustered distribution (Fig. 1E). The clustered distribution of hybrid depression indicates its preferential occurrence in long recombination deserts.

We identified four large genomic regions associated with hybrid depression, all located within recombination deserts on chromosomes 1, 3, 4, and 8. For instance, on chromosomes 1 and 8, pronounced hybrid depression effects were observed across extensive genomic fragments spanning chr1 : 120.9–164.3 Mb and chr8 : 33.1–85.7Mb (Fig. S7). In contrast, inbreeding depression was confined to relatively shorter genomic fragments, with the longest on chromosomes 6 and 15 (Fig. S7). Both hybrid and inbreeding depression hotspots were found to lack high recombination rates, yet they exhibited distinct patterns of *F*_ST_ between the LW and MIN pigs. The observed hybrid depression likely contributes to reproductive barriers, as exemplified by a hybrid depression region on chromosome 3 (39.0–41.5 Mb) that overlaps with a locus of hybrid incompatibility previously implicated in the nascent speciation of Eurasian pigs [22].

Recombination is expected to enhance the efficiency of natural selection [12], leading to heterogeneous evolutionary rates across the genome that correlate with varying hybrid effects. Consistently, genomic analysis demonstrated that hybrid vigor windows had the highest SNP density (1711 SNPs per window), which was significantly greater than both inbreeding depression (1632 SNPs per window; t-test, *P* = 2.91 × 10⁻⁹) and hybrid depression windows (1554 SNPs per window; *P* < 2.20 × 10⁻^16^) (Fig. S8). A positive correlation (*r* = 0.90, *P* = 5.87 × 10^−8^) was further observed between the SNP density and λ (Fig. S8), indicating that the rate of genome evolution has been significantly affected by the recombination-mediated hybrid effect variation. Permutation test showed that the higher SNP density difference in the hybrid vigor regions is unlikely to be caused by random events (*P* < 1.0 × 10^−6^; 1.0 × 10^6^ replicates; Fig. S8).

### Sex difference in hybrid effects and strong hybrid female depression

**Figure 2.**
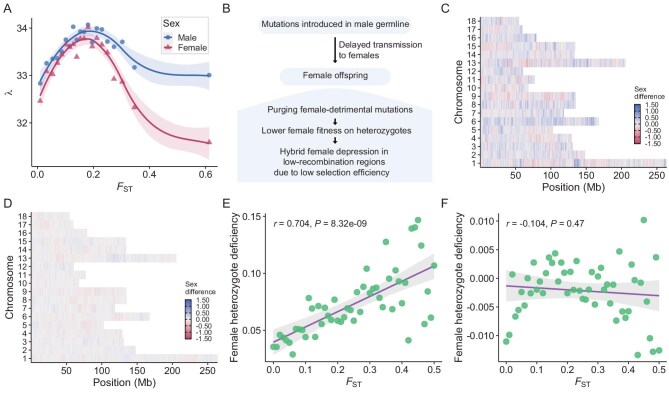
Sex difference in hybrid effects and female-detrimental effects in low-recombination hybrid depression regions. (A) Constitutively lower λ values for hybrid effects in females compared to in males. (B) A hypothesis for female-detrimental hybrid effects in male-driven evolution and hybrid female depression caused by low recombination rates in females. (C) Deficiency of female heterozygotes for rare SNPs (MAF ≤ 0.05) revealed by the sex difference in heterozygote ratio (HR) in the F2 population, calculated as [HR(male)—HR(female)]/[(HR(male) + HR(female))/2]. Blue and red denote female and male heterozygote deficiencies, respectively. (D) Plot of sex-biased heterozygote deficiency for common SNPs (MAF > 0.05). (E) Positive correlation between female heterozygote deficiency for rare SNPs and *F*_ST_ and the highest level of female heterozygote deficiency in hybrid depression regions (*F*_ST_ > 0.26). (F) Analysis of common SNPs reveals no enrichment of female heterozygote deficiency in hybrid depression regions.

Given the role of recombination in shaping hybrid effects and the known differences in recombination rates between sexes, we expected that hybrid effects would exhibit sex-specific differences, exerting a comprehensive influence on fitness outcomes. To explore the sex difference, we compared the λ distribution of the 135 traits in both sexes. Our analysis revealed a hump-shaped distribution of λ in both males and females (Fig. 2A), yet with a significant sex difference that females consistently displayed lower λ values than males across all three hybrid effect classes (Wilcoxon signed-rank test, *P* = 2.15 × 10⁻⁵). This suggests that the females may experience low levels of beneficial hybrid outcome in the hybridization of Eurasian pigs. Consistent with this finding, we observed a slightly skewed female ratio (49.13%) in the LW-MIN F2 population, deviating from the expected 1 : 1 equilibrium [32,39]. This pattern was further corroborated in two additional F2 populations developed from the East Asian Diannan small-ear (DNSE) pig ♂ × LW ♀ and LW ♂ × DNSE ♀, with the female ratios being 47.54% (*n_F2_* = 2215) and 45.36% (*n_F2_* = 388) respectively.

The most striking sex difference occurred at the right tail of the hybrid effect distribution, where females displayed a more substantial decline in λ than males, especially in genomic regions with *F*_ST_ > 0.5. This observation highlights a preferential evolution of hybrid female depressions. These high-divergence regions (*F*_ST_ > 0.5) encompassed 157 windows (Table S5) exhibiting extremely low recombination rates in both sexes. Of these windows, 64 were from the chromosome 8 (Fig. 1E). Interestingly, the 157 windows demonstrated sexually antagonistic transmission of recombination from F1 parents to F2 offspring. Among 294 F2 males, 18 paternal and 9 maternal recombination breakpoints were inherited. In contrast, 284 F2 females inherited 12 paternal but 20 maternal recombination breakpoints (Fisher’s exact test, *P* = 3.70 × 10^−2^). The skewed maternal inheritance percentages (33.33% in males *vs.* 62.50% in females) differed from the genome-wide averages (56.75% and 57.15% for F2 males and females, respectively). On the 64 windows of chromosome 8, the F2 females inherited 6 maternal and 0 paternal recombination breakpoints, compared to 1 maternal and 2 paternal recombination breakpoints inherited by F2 males (*P* = 0.08). This father-to-son and mother-to-daughter bias in recombination breakpoint transmission supports a model of sexually antagonistic evolution [40–42], which may partially alleviate hybrid depression in each sex. However, this sex-specific transmission pattern alone cannot fully explain the pronounced female λ reduction at the right tail, particularly given a higher total amount of recombination in the maternal genomes of the F2.

Combining the sexually antagonistic transmission of recombination breakpoints with the well-established role of recombination in purging deleterious mutations [11,43], we infer that the lower female λ values in the right tail may partially stem from asymmetric deleterious effects of genomic mutations introduced in females versus males. It is well-documented that new mutations are male-biased in origin and arise preferentially in the male germline [44–47]. New mutations originating in the male germline are already exposed to natural selection in males during spermatogenesis, and thus experience delayed selection in female offspring. This delayed selection in females means that they may have a shorter selection window to purge female-detrimental mutations, an effect expected to be strongest in recombination coldspots within hybrid depression regions where selection efficacy is reduced (Fig. 2B). Rare SNPs, owing to their relatively recent mutational origin, provide an excellent proxy for testing this hypothesis. To investigate the putative mutational impact on females, we performed whole-genome resequencing on 521 F2 individuals (265 males and 256 females) and compared the heterozygote distribution of SNPs between F2 males and females after applying strict quality control to the variant calls (see Materials and Methods). We found that among F2 females, heterozygote deficiency exists for rare SNPs with minor allele frequency (MAF) ≤ 0.05 (Fig. 2C). In total, 13 200 100-kb windows exhibited higher overall heterozygote frequency of rare SNPs in males, compared to 8849 windows with higher frequency in females (*P* < 1.0 × 10^−22^; binomial test; Fig. S9). Conversely, for common SNPs (MAF > 0.05), no obvious difference in heterozygote frequency was observed between the sexes (Fig. 2D), with 11 079 and 10 970 windows showing higher overall heterozygote frequencies in males and females respectively (*P* = 0.467; Fig. S9). We also detected a strong positive correlation between heterozygote deficiency of rare SNPs in females and window-based *F*_ST_ values (*r* = 0.70, *P* = 8.32 × 10^−9^) (Fig. 2E). No such correlation was evident for common SNPs (*r* = −0.10, *P* = 0.47) (Fig. 2F). The highest female heterozygote deficiency for rare SNPs in highly differentiated genomic regions indicates that these new/rare SNPs tend to have deleterious effects in females. This provides a potential explanation for the strong hybrid female depression observed: hybrid depression regions are clustered in low-recombination genomic regions, where the efficacy of selection to purge female-deleterious variants is reduced (Fig. 1E).

### Genotype-phenotype coevolution and heterozygote advantage in λ peak

**Figure 3.**
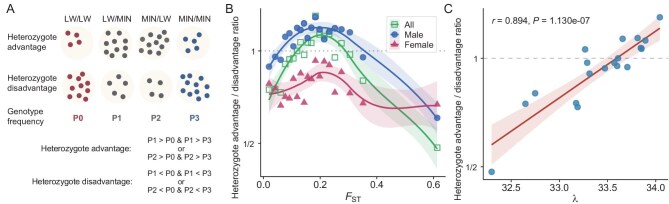
The genotype-phenotype coevolution revealed by a hump-shaped distribution of heterozygote advantage/disadvantage ratio in the LW-MIN F2 population. (A) Schematic diagram of identifying heterozygote advantage by comparing the frequency between heterozygous and homozygous genotypes of 100-kb windows in the F2 population. The heterozygote advantage/disadvantage ratio in a *F*_ST_ bin is the ratio of the number of windows with heterozygote advantage to the number of windows with heterozygote disadvantage. (B) Cooccurrence of the λ peak in phenotypic space (Fig. 1B) and the heterozygote advantage in genotypic space revealed by a hump-shaped distribution of the heterozygote advantage/disadvantage ratio across the window bins grouped by *F*_ST_. The horizontal line indicates a balance with equal heterozygote and homozygote frequencies. (C) A positive correlation observed between the phenotypic λ and the heterozygote advantage/disadvantage ratio.

The link between hybrid vigor (characterized by the λ peak in phenotypic evolution) and heterozygote advantage at the genotypic level is a fundamental question in hybridization research. This relationship can fundamentally shape genome evolution by reshaping genotype frequency distributions in hybrid populations. We hypothesized that the favorable hybrid effects marked by the λ peak in phenotypic evolution would confer relatively higher fitness to heterozygous genotypes during genome evolution. To investigate this genotype-phenotype coevolution, we compared genotype frequencies in our F2 resource population, and quantified the relative fitness of heterozygous genotypes versus their homozygous counterparts across genomic regions showing distinct hybrid effects (Fig. 3A). To align with the phenotypic λ estimation framework that compares heterozygotes against homozygotes, we defined the heterozygote advantage/disadvantage ratio as the ratio of genomic windows with heterozygote advantage to those with heterozygote disadvantage. This ratio provides a direct, quantitative measure of the relative fitness of heterozygotes compared with homozygotes across the genome.

Consistently, the heterozygote advantage/disadvantage ratio exhibited a hump-shaped distribution across the *F*_ST_ bins (Fig. 3B; Table S6), closely matching the hybrid vigor pattern observed in phenotypic evolution (Fig. 1B). Notably, this ratio showed a significantly positive correlation with λ (Fig. 3C) (*r* = 0.89, *P* = 1.13 × 10⁻⁷), suggesting coevolution between genomic heterozygote advantage and phenotypic hybrid vigor. Sex-specific analysis revealed that males exhibited greater heterozygote advantage (in 51.84% of windows) than females (in 43.55%), highly consistent with the observation of constitutively lower λ in females (Fig. 2A). To test whether the elevated heterozygote advantage/disadvantage ratio in the hybrid vigor regions arises from factors unrelated to the hybrid effects, we conducted permutation tests assuming that genomic windows with distinct heterozygote advantage/disadvantage ratios were randomly distributed across the three hybrid effect types. The result showed that this increase in the hybrid vigor regions is unlikely to be caused by random events (All: *P* < 1.0 × 10^−6^; Male: *P* < 1.0 × 10^−6^; Female: *P* = 1.0 × 10^−6^; 1.0 × 10^6^ replicates; Fig. S10). The peak in the female genomic heterozygote advantage/disadvantage ratio indicates enhanced relative fitness of heterozygotes in genomic regions associated with the phenotypic λ peak, even against the overall backdrop of this ratio in females.

### Difference between evolutionary hybrid vigor and quantitative mid-parent heterosis

A central question in hybridization research is how hybrid effects observed from an evolutionary perspective relate to MPH, a metric widely used in quantitative studies. The MPH quantifies the phenotypic deviation of hybrids from the mid-parent value, capturing non-additive genetic effects that contrast with Fisher’s additive genetic variance in adaptation [32]. In contrast, our study evaluates the hybrid effects by converting phenotypic changes into evolutionary metrics: the relative probability of beneficial phenotypic changes and corresponding allelic spread patterns of genomic heterozygotes in the F2 population. To reconcile these differing frameworks, we mapped MPH values to genomic regions exhibiting distinct evolutionary hybrid effects in our study. Since the 135 traits were not available for the F0 founders, we calculated a new phenotypic statistic, MPH’ as a biological approximation for the MPH with difference, by quantifying the phenotypic deviation of F2 heterozygotes (LW/MIN or MIN/LW) from the phenotypic mean of the F2 homozygotes (LW/LW and MIN/MIN).

**Figure 4.**
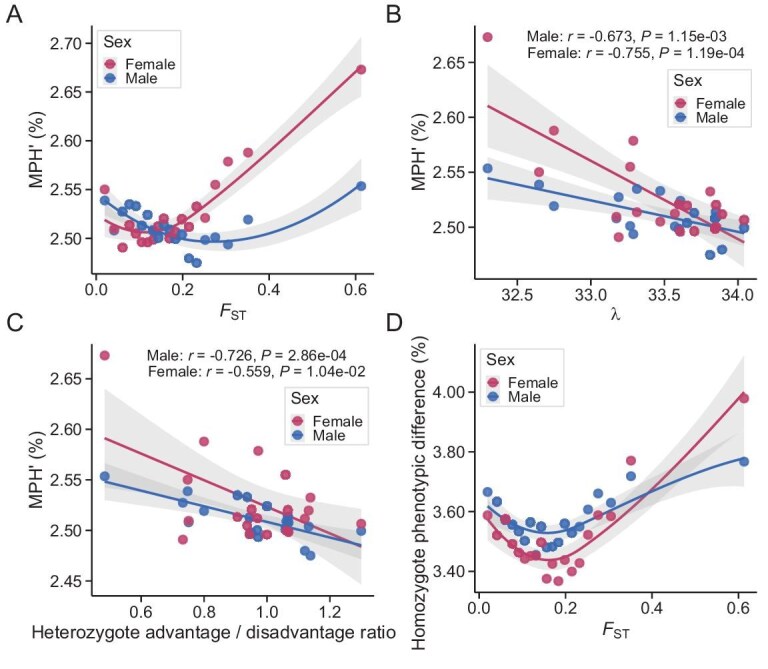
Distribution of MPH’ and its negative correlation with beneficial hybrid effects. (A) Occurrence of higher levels of MPH’ in the evolutionary hybrid depression regions (*F*_ST_ > 0.26). The MPH’ abbreviates for an approximation for mid-parent heterosis. (B) Negative correlation observed between the MPH’ and the evolutionary λ parameter. (C) Negative correlation observed between MPH’ and the heterozygote advantage/disadvantage ratio. (D) Distribution of phenotypic difference between the LW/LW and MIN/MIN homozygotes with lowest difference in the evolutionary hybrid vigor regions and highest in the hybrid depression regions.

As a result, a steady increase in MPH’ was observed in both sexes within hybrid depression regions compared to inbreeding depression and hybrid vigor regions (Fig. 4), highlighting its distinct expression pattern in crosses between highly differentiated parents. The highest MPH’ values, averaged across 135 traits, reached 2.67% in females and 2.55% in males at the extreme right end of the hybrid effect spectrum. Significant negative correlations were observed between MPH’ and λ (males: *r* = −0.67, *P* = 1.15 × 10^−3^; females: *r* = −0.76, *P* = 1.19 × 10^−4^) (Fig. 4B), as well as between MPH’ and heterozygote advantage/disadvantage ratio (males: *r* = −0.73, *P* = 2.86 × 10^−4^; females: *r* = −0.56, *P* = 1.04 × 10^−2^) (Fig. 4C). Given the increasing tendency of being beneficial for heterozygotes with high λ in the FGM and high advantage/disadvantage ratio in the F2 population, the strong negative correlations suggest the high MPH’ tends to reflect evolutionary heterozygote disadvantage as compared to homozygotes in both phenotypic evolution and population genetics. Interestingly, a U-shaped distribution of phenotypic differences between LW/LW and MIN/MIN homozygotes was observed in both sexes across the *F*_ST_ bins (Fig. 4D). These homozygote phenotypic differences were most pronounced in hybrid depression regions, particularly for females, and minimal in hybrid vigor regions. Collectively, these results demonstrate that quantitative MPH’ may have elevated levels in hybrid depression regions, when the hybridization combines highly divergent parental alleles with large phenotypic difference.

## DISCUSSION

This study revealed substantial variation in hybrid effects across the Eurasian pig genome. Following comprehensive analysis of phenotypic variation across 135 traits in the LW-MIN F2 population, we delineated the nuanced dynamics within an evolutionary continuum spanning from inbreeding depression through hybrid vigor to hybrid depression in the Eurasian pig genome. Our findings establish recombination as a mediator of hybrid effect transition through its interplay with genomic differentiation. The co-occurrence of advantageous effects in high-recombination genomic regions across phenotypic and genotypic spaces suggests an integrated process of genotype-phenotype coevolution during hybridization, thereby resolving longstanding debates regarding hybridization’s evolutionary impact. Furthermore, our study reveals comprehensive sex differences in hybrid effects and highlights fundamental conceptual divergences between quantitative genetics and evolutionary biology in understanding hybrid effects.

The transition of hybrid effects across the Eurasian pig genome suggests that recombination mediates a heterogeneous pace of genome evolution, challenging the conventional dichotomous classification of hybridization outcomes as either hybrid vigor or hybrid incompatibilities. Our results demonstrate that the process of within-genome evolution—which generates heterogeneous hybrid effects—is highly dynamic, providing new insight into how recombination shapes a continuous spectrum of hybrid effects across distinct genomic regions. While the concept of an optimal mating distance was proposed previously [14], our findings show that beneficial hybrid effects preferentially accumulate in genomic regions with high recombination rates, rather than being randomly distributed across the genome in any given hybridization event. Although exploring optimal mating distance undoubtedly yields valuable insights into hybridization outcomes [16,35], we emphasize that the observed optimal mating distance is likely dynamic: recombination occurs every generation, and its cumulative effects shape genomic diversity and hybrid effects over long-term evolution. We therefore propose that future hybrid genetics research should prioritize comparative analyses of hybrid effects between recombination hotspots and coldspots.

Our experiment further yields the robust finding that, at an appropriate level of parental divergence, the most beneficial hybrid effects do not typically originate from genomic regions with the highest degree of genetic differentiation between parental genomes. While transgressive segregation has been observed in specific traits of hybrids by studies on hybrid adaptation especially when the hybrid genome become genetically stabilized [2,6], this established model holds that adaptive evolution in hybrids relies on complementary effects of distinct loci fixed for alternative alleles with opposite directional effects in the two parental species. In contrast to this framework focused on the later stabilization stage of hybrid genomes, our observations emphasize that the most beneficial effects across a broad spectrum of phenotypes in the first few generations of hybridization are primarily associated with recombination-mediated hybrid effects. This mechanism may represent a common general pathway that allows hybrids to adapt effectively before their genomes reach genetic stabilization.

Introducing the λ parameter for describing phenotypic variation offers a genome-wide perspective on hybrid effects and establishes a novel framework for investigating genomic heterogeneity in hybrid evolution. Crucially, λ can be applied to multi-dimensional empirical data to untangle the complex interplay between genetic recombination, genome evolution, and phenotypic evolution, representing a key conceptual advance since the impact of recombination on genome evolution (via purging deleterious mutations) has been characterized theoretically [12]. The strong correlations observed between empirical λ values and other common genomic metrics (including recombination rate, genetic differentiation, SNP density, and MPH’) highlight that the new genome-wide summary metric has fundamental value for unraveling the coevolutionary dynamics of genomes and phenotypes. It may also serve as a new conceptual bridge to disentangle the evolutionary links between these established metrics. Despite these important advances, applying λ to hybrid studies still faces several notable challenges. First, estimating λ generally requires large phenotypic trait datasets, with accuracy directly tied to both sample size and the number of traits analyzed. Second, λ currently has limited capacity to elucidate how covariation among multiple traits drives hybrid evolution. Third, as an aggregate metric calculated over genomic regions rather than at the level of individual variants, λ is not well-suited for dissecting the effects of individual specific loci.

The sex difference in λ distributions across different levels of genetic differentiation also provides novel insights into the differential evolution of the two sexes during hybridization. The consistently lower λ in females, combined with the most pronounced drop in λ within recombination deserts (hybrid depression regions), points to a distinct role of recombination in female-mediated hybrid evolution. Although this link remains untested in the present study, the well-documented biased distribution of recombination in females [30,31] could share an evolutionary connection with the reduced λ values we observe here. While we hypothesized that new mutations may exert slightly deleterious effects on females in the context of male-driven evolution, this does not exclude other evolutionary scenarios that could explain the female-specific λ drop in hybrid depression regions. Drawing from our observation of father-to-son and mother-to-daughter transmission bias of recombination breakpoints in part of hybrid depression regions, future studies disentangling the putative mutation-recombination-selection balance between sexes may reveal more subtle population dynamics during the early stages of hybrid population evolution.

Collectively, our findings provide comprehensive experimental evidence elucidating the impact of hybridization on genome and phenotype evolution in Eurasian domestic pig crosses. These results underscore the necessity for careful consideration when exploring hybrid effects across different hybridization scenarios, particularly in evolutionary studies addressing hybrid fitness variation and in breeding practices aimed at enhancing hybrid productive performance.

## Supplementary Material

nwag322_Supplemental_Files

## MATERIALS AND METHODS

Detailed Materials and Methods are available in the Supplementary data.

## References

[1] Barton NH . The role of hybridization in evolution. Mol Ecol 2001; 10: 551–68. 10.1046/j.1365-294x.2001.01216.x 11298968

[2] Seehausen O . Hybridization and adaptive radiation. Trends Ecol Evol 2004; 19: 198–207. 10.1016/j.tree.2004.01.003 16701254

[3] Taylor SA, Larson EL. Insights from genomes into the evolutionary importance and prevalence of hybridization in nature. Nat Ecol Evol 2019; 3: 170–7. 10.1038/s41559-018-0777-y 30697003

[4] Mallet J . Hybrid speciation. Nature 2007; 446: 279–83. 10.1038/nature05706 17361174

[5] Rieseberg LH . Hybrid origins of plant species. Annu Rev Ecol Syst 1997; 28: 359–89. 10.1146/annurev.ecolsys.28.1.359

[6] Rieseberg LH, Raymond O, Rosenthal DM et al. Major ecological transitions in wild sunflowers facilitated by hybridization. Science 2003; 301: 1211–6. 10.1126/science.1086949 12907807

[7] Powell DL, Garcia-Olazabal M, Keegan M et al. Natural hybridization reveals incompatible alleles that cause melanoma in swordtail fish. Science 2020; 368: 731–6. 10.1126/science.aba5216 32409469 PMC8074799

[8] Coyne JA, Orr HA. Speciation. Sunderland (MA): Sinauer Associates, 2004.

[9] Wu CI, Palopoli MF. Genetics of postmating reproductive isolation in animals. Annu Rev Genet 1994; 28: 283–308. 10.1146/annurev.ge.28.120194.001435 7893128

[10] Kondrashov AS . Deleterious mutations and the evolution of sexual reproduction. Nature 1988; 336: 435–40. 10.1038/336435a0 3057385

[11] Felsenstein J . The evolutionary advantage of recombination. Genetics 1974; 78: 737–56. 10.1093/genetics/78.2.737 4448362 PMC1213231

[12] Hill WG, Robertson A. The effect of linkage on limits to artificial selection. Genet Res 1966; 8: 269–94. 10.1017/S0016672300010156 5980116

[13] Barton NH, Charlesworth B. Why sex and recombination? Science 1998; 281: 1986–90. 10.1126/science.281.5385.1986 9748151

[14] Bateson P . Sexual imprinting and optimal outbreeding. Nature 1978; 273: 659–60. 10.1038/273659a0 661972

[15] Lynch M . The genetic interpretation of inbreeding depression and outbreeding depression. Evolution 1991; 45: 622–9. 10.1111/j.1558-5646.1991.tb04333.x 28568822

[16] Wei X, Zhang J. The optimal mating distance resulting from heterosis and genetic incompatibility. Sci Adv 2018; 4: eaau5518. 10.1126/sciadv.aau5518 30417098 PMC6221538

[17] Huang X, Yang S, Gong J et al. Genomic architecture of heterosis for yield traits in rice. Nature 2016; 537: 629–33. 10.1038/nature19760 27602511

[18] Yang M, Wang X, Ren D et al. Genomic architecture of biomass heterosis in Arabidopsis. Proc Natl Acad Sci USA 2017; 114: 8101–6. 10.1073/pnas.1705423114 28696287 PMC5544317

[19] Scally A, Dutheil JY, Hillier LW et al. Insights into hominid evolution from the gorilla genome sequence. Nature 2012; 483: 169–75. 10.1038/nature10842 22398555 PMC3303130

[20] Rivas-Gonzalez I, Rousselle M, Li F et al. Pervasive incomplete lineage sorting illuminates speciation and selection in primates. Science 2023; 380: eabn4409. 10.1126/science.abn4409 37262154

[21] Tao L, Wang LG, Adeola AC et al. Associations of autozygosity with economic important traits in a cross of Eurasian pigs. J Genet Genomics 2023; 50: 216–20. 10.1016/j.jgg.2022.09.002 36152906

[22] Xie HB, Wang LG, Fan CY et al. Genetic architecture underlying nascent speciation—the evolution of Eurasian pigs under domestication. Mol Biol Evol 2021; 38: 3556–66. 10.1093/molbev/msab117 33892509 PMC8382894

[23] Saura M, Fernandez A, Varona L et al. Detecting inbreeding depression for reproductive traits in Iberian pigs using genome-wide data. Genet Sel Evol 2015; 47: 1. 10.1186/s12711-014-0081-5 25595431 PMC4297446

[24] Larson G, Dobney K, Albarella U et al. Worldwide phylogeography of wild boar reveals multiple centers of pig domestication. Science 2005; 307: 1618–21. 10.1126/science.1106927 15761152

[25] Wu GS, Yao YG, Qu KX et al. Population phylogenomic analysis of mitochondrial DNA in wild boars and domestic pigs revealed multiple domestication events in East Asia. Genome Biol 2007; 8: R245. 10.1186/gb-2007-8-11-r245 18021448 PMC2258183

[26] Groenen MA, Archibald AL, Uenishi H et al. Analyses of pig genomes provide insight into porcine demography and evolution. Nature 2012; 491: 393–8. 10.1038/nature11622 23151582 PMC3566564

[27] China National Commission of Animal Genetic Resources . Animal Genetic Resources in China: Pigs. Beijing: China Agriculture Press, 2011.

[28] Frantz LA, Schraiber JG, Madsen O et al. Evidence of long-term gene flow and selection during domestication from analyses of Eurasian wild and domestic pig genomes. Nat Genet 2015; 47: 1141–8. 10.1038/ng.3394 26323058

[29] Frantz L, Meijaard E, Gongora J et al. The evolution of Suidae. Annu Rev Anim Biosci 2016; 4: 61–85. 10.1146/annurev-animal-021815-111155 26526544

[30] Kong A, Gudbjartsson DF, Sainz J et al. A high-resolution recombination map of the human genome. Nat Genet 2002; 31: 241–7. 10.1038/ng917 12053178

[31] Kong A, Thorleifsson G, Gudbjartsson DF et al. Fine-scale recombination rate differences between sexes, populations and individuals. Nature 2010; 467: 1099. 10.1038/nature09525 20981099

[32] Fisher RA . The Genetical Theory of Natural Selection. Oxford: Oxford University Press, 1930. 10.5962/bhl.title.27468

[33] Orr HA . The population genetics of adaptation: the distribution of factors fixed during adaptive evolution. Evolution 1998; 52: 935–49. 10.1111/j.1558-5646.1998.tb01823.x 28565213

[34] Connallon T, Hodgins KA. Allen Orr and the genetics of adaptation. Evolution 2021; 75: 2624–40. 10.1111/evo.14372 34606622

[35] Moll RH, Lonnquist JH, Fortuno JV et al. The relationship of heterosis and genetic divergence in maize. Genetics 1965; 52: 139–44. 10.1093/genetics/52.1.139 17248265 PMC1210832

[36] Lippman ZB, Zamir D. Heterosis: revisiting the magic. Trends Genet 2007; 23: 60–6. 10.1016/j.tig.2006.12.006 17188398

[37] Chen ZJ . Genomic and epigenetic insights into the molecular bases of heterosis. Nat Rev Genet 2013; 14: 471–82. 10.1038/nrg3503 23752794

[38] Kimura M . The Neutral Theory of Molecular Evolution. Cambridge: Cambridge University Press, 1983. 10.1017/CBO9780511623486

[39] Hamilton WD . Extraordinary sex ratios. A sex-ratio theory for sex linkage and inbreeding has new implications in cytogenetics and entomology. Science 1967; 156: 477–88. 10.1126/science.156.3774.477 6021675

[40] Rice WR . Sex chromosomes and the evolution of sexual dimorphism. Evolution 1984; 38: 735–42. 10.1111/j.1558-5646.1984.tb00346.x 28555827

[41] Foerster K, Coulson T, Sheldon BC et al. Sexually antagonistic genetic variation for fitness in red deer. Nature 2007; 447: 1107–10. 10.1038/nature05912 17597758

[42] Eyer PA, Blumenfeld AJ, Vargo EL. Sexually antagonistic selection promotes genetic divergence between males and females in an ant. Proc Natl Acad Sci USA 2019; 116: 24157–63. 10.1073/pnas.1906568116 31719204 PMC6883821

[43] Muller HJ . The relation of recombination to mutational advance. Mutat Res 1964; 106: 2–9. 10.1016/0027-5107(64)90047-8 14195748

[44] Bergeron LA, Besenbacher S, Zheng J et al. Evolution of the germline mutation rate across vertebrates. Nature 2023; 615: 285–91. 10.1038/s41586-023-05752-y 36859541 PMC9995274

[45] Li WH, Yi S, Makova K. Male-driven evolution. Curr Opin Genet Dev 2002; 12: 650–6. 10.1016/S0959-437X(02)00354-4 12433577

[46] Ellegren H, Fridolfsson AK. Male-driven evolution of DNA sequences in birds. Nat Genet 1997; 17: 182–4. 10.1038/ng1097-182 9326938

[47] Makova KD, Li WH. Strong male-driven evolution of DNA sequences in humans and apes. Nature 2002; 416: 624–6. 10.1038/416624a 11948348

