# Supplementary material for: Genetic recombination shapes complex hybrid effects across the pig genome
Source: Natl Sci Rev. 2026 May 28;13(13):nwag322. doi: 10.1093/nsr/nwag322 (PMC13339142; doi:10.1093/nsr/nwag322)
Supplement: nwag322_Supplemental_Files [file nwag322_supplemental_files.zip › Supplementary data.docx]

**Supplementary Information**

**Genetic recombination shapes complex hybrid effects across the pig genome**

Hai-Bing Xie^1†*^, Zi-Qin Huang^1,2†^, Li-Gang Wang^3†^, Long-Chao Zhang^3†^, Shu-Shu Yan^9^, Jia-Kun Deng^1^, Adeniyi C. Adeola^1^, Qing-Long Li^9^, Lin Tao^1,7,8^, Kui Li^5^, Shu-Hong Zhao^6^, Zhao-Bang Zeng^4^, Li-Xian Wang^3*^, Ya-Ping Zhang^1,9*^

1 State Key Laboratory of Genetic Evolution & Animal Models and Yunnan Key Laboratory of Molecular Biology of Domestic Animals, Kunming Institute of Zoology, Chinese Academy of Sciences, Kunming 650223, China

2 Division of Life Sciences and Medicine, University of Science and Technology of China, Hefei 230026, China

3 Institute of Animal Science, Chinese Academy of Agricultural Sciences, Beijing 100193, China

4 Bioinformatics Research Center, Department of Horticultural Science, North Carolina State University, Raleigh, NC, USA

5 Shenzhen Branch, Guangdong Laboratory of Lingnan Modern Agriculture, Key Laboratory of Livestock and Poultry Multi-omics of MARA, Agricultural Genomics Institute at Shenzhen, Chinese Academy of Agricultural Sciences, Shenzhen, 518124, China

6 Key Laboratory of Agricultural Animal Genetics, Breeding and Reproduction, Ministry of Education & Key Laboratory of Pig Genetics and Breeding, Ministry of Agriculture and Rural Affairs, Huazhong Agricultural University, Wuhan, 430070, China

7 Kunming College of Life Science, University of Chinese Academy of Sciences, Kunming 650204, China

8 University of Chinese Academy of Sciences, Beijing 100049, China

9 Bio-X Center for Interdisciplinary Innovation, School of Ecology and Environmental Science, Yunnan University, Kunming 650500, PR China

**This file includes:**

Materials and Methods

Figures S1-S10

Tables S1-S6

References (1-8)

**Materials and Methods**

**Ethics**

This study was approved by the Animal Care and Ethics Committee of Kunming Institute of Zoology, Chinese Academy of Sciences (Approval ID: IACUC-OE-2021-11-002).

**Experimental populations**

To explore the hybrid effects across the genome, we used the Eurasian domestic pigs as a model to explore the beneficial and detrimental effects that are introduced by hybridization. The first family was developed by a cross between the European Large White (LW) and East Asian Min (MIN) pigs. The LW-MIN family was constructed using five LW males and 15 MIN sows as the F0 founders. A total of nine F1 males and 45 F1 females was chosen for producing the F2. All the F2 were raised after birth without any human-mediated selection for productive performance. The F2 consisted of 294 males and 284 females at 240 ± 7 days after birth. The sex ratio was calculated on the 240 days. The second and the third families were developed from the crosses between the LW and the East Asian Diannan-small ear (DNSE) pigs. The second was founded by five DNSE males and 23 LW females (n_F2_ = 2,215), and the third was developed from the cross of six LW males and 18 DNSE females (n_F2_ = 388). The sex ratios in the DNSE-LW and LW-DNSE families were calculated on the F2 offspring at birth.

We examined the hybrid effects in the LW-MIN family by exploring their impacts on the phenotypic variation and the population genetics in the F2 generation. To obtain a comprehensive representative of the physiologies underlying the development, the phenotypes for F2 individuals in the LW-MIN family were collected on 135 traits (**Table S2**), spanning the muscle fiber type/area, body size/height/weight, fat deposition, skeletal size/weight, organ weights, skin color and ear shape. For the body weight traits, phenotypic data were collected at birth (0d), 30d, 60d, 120d, 180d, and 240d. For the remaining traits, the data were collected at 240±7 days after birth.

**Haplotype inference and recombination breakpoint identification**

All the LW-MIN family members were genotyped using the Illumina PorcineSNP60 BeadChip, which employed 62,163 SNPs across the pig genome. All the following analysis was based on the pig genome build Sscrofa 11.1. The autosomal haplotypes were inferred using the chip data with the SHAPEIT2 software [1], and the pedigree information was included to correct the phasing errors. To fully reconstruct the transmission path of autosomal fragments from F0 to F1 and finally to F2, the haplotypes in each F2 were resolved as paternal and maternal according to their sequence similarity to the four haplotypes in the F1 parents, and similarly the two autosomal haplotypes in each F1 were further resolved in the F0 to F1 transmission. Combining the two steps, each of the F2 haplotypes can be determined as either an autosome that may carry the LW or MIN allele when no recombination events was involved in the F1 to F2 transmission, or a recombinant autosome with LW and MIN alleles in different loci when recombination events occurred. The recombination breakpoints were determined between two adjacent SNPs that carry the alleles with different breed origins. The analysis was conducted on 100-kb genomic windows and all the window-wise analyses in this study were performed on the same dataset of 100-kb windows. The reconstruction of transmission path and the identification of the recombination events in the F2 haplotypes were implemented in R scripts. For the details, please refer to our GitHub repository at https://github.com/xiehb-evolution/hybrid-effects/.

**Hybrid effect analysis**

To explore the variation of hybrid effects, we compared the phenotypic difference between the two homozygotes (MIN/MIN and LW/LW) and the two heterozygotes (MIN/LW and LW/MIN) in each of the 100-kb windows. The hybrid effect was defined as the homozygote-heterozygote difference in the phenotypic means of the considered genotypes scaled to the population mean of a sex. To obtain the phenotypic mean of a genotype at a 100-kb window, the F2 samples were grouped according to their genotypes in each of the autosomal windows. For a specific 100-kb window, F2 samples were not included in the following analysis if they contained recombination events in either paternal or maternal genomes, because the genotype of that window can not be uniquely identified for those samples. On each window, the homozygote-heterozygote phenotypic difference was calculated four times, representing the MIN/MIN-MIN/LW, MIN/MIN-LW/MIN, LW/LW-MIN/LW, and the LW/LW-LW/MIN genotype comparisons. The phenotypic mean for a subset of samples carrying a same genotype on a 100-kb window was calculated. The calculation was conducted separately in F2 males and females. The hybrid effect size is calculated as following:

$$hybrid effect size=\frac{|phenotype\left( \mathrm{heterozygote} \right)-phenotype\left( \mathrm{homozygote} \right)|}{phenotype(sex)}$$

Here, for each of the 135 traits, the *phenotype(homozygote)* and *phenotype(heterozygote)* represent the phenotypic means of F2 individuals from a sex carrying specific homozygous and heterozygous genotypes in a 100-kb window, and the *phenotype(sex)* is the phenotypic mean of a sex irrespective of the genotypes.

To quantify the relative probability of being beneficial, we applied the analysis of hybrid effects in the framework of Fisher’s geometric model (FGM) [2] by putting the homozygotes as wild types and the heterozygotes as mutants. In the FGM, a new mutation of large effect size tends to be harmful due to its tendency to cause large deviation from a fitness optimum as compared to the wild type. The probability of being beneficial would be highest at 0.5 if the mutation size approaches zero and decrease along with the mutation size increase because large effect size of a mutation tends to make the mutation exhibit a larger distance to the optimum as compared to the wild type [2]. Differently, we did not define the optima of phenotypes for the FGM, but rather quantify the homozygote-heterozygote phenotypic difference that was normalized on a scale of the phenotypic mean of a trait in each sex. The homozygote-heterozygote phenotypic difference was calculated on each 100-kb windows and in each of the 135 traits. We assumed the homozygotes to be wild types and the heterozygotes introduced by hybridization to be mutant types in the FGM, the relative beneficial probability of hybrid effects would be determined by the homozygote-heterozygote phenotypic difference. The homozygote-heterozygote phenotypic difference for all the 135 traits were combined to infer the hybrid effect exponential distribution. The hybrid effect size distribution and the λ parameter estimation was conducted in each sex using the ‘fitdistr’ function in the *fitdistrplus* R package[3]. All the λ was estimated in F2 males and F2 females independently. In the whole-genome analysis, the female λ was estimated using the phenotypic data of F2 females, and the male λ was estimated using the phenotypic data of F2 males. The λ identified here is an important measure of the relative probability of being beneficial for hybrid effects (see below). The detailed scripts for the calculation were supplied under the GitHub repository.

We chose the λ as an indicator to scale the probability of beneficial hybrid effects. In the exponential distribution, λ equals the reciprocal of the expected hybrid effect size r (λ = 1/r). According to FGM, the probability of a beneficial effect is given by P_beneficial_ = 1 – Ф(*x*), where the term *x* is defined as $x =\frac{r\sqrt{n}}{d}$[4]. In this context, *d* represents the diameter of the "sphere" in FGM, which corresponds to twice the distance from the current state to the phenotypic optimum, and *n* denotes the dimensionality of the phenotypic space (practically, *n* = 1 for individual trait analysis). Here, Ф(*x*) is the cumulative distribution function (CDF) of the standard normal distribution. Mathematically, P_beneficial_ represents the cumulative probability of the right tail starting from the cutoff *x*. Consequently, a larger λ value (reflecting a smaller *r*) shifts the cutoff *x* to the left, thereby monotonically increasing P_beneficial_.

To explore the transition of hybrid effects from inbreeding depression, hybrid vigor, and to hybrid depression, the LW-MIN founder pigs were resequenced using the HiSeq2000 platform with libraries of 500-bp inserts. The genomic reads were mapped to the Duroc reference genome[5] using the Burrows-Wheeler Aligner (BWA)[6], and SAMtools[7] was used to sort/merge the BAM files and remove potential PCR duplications. To reduce the level of misalignment of short reads from paralogous regions, the secondary mappings of genomic reads were removed for the following analysis. Genome Analysis Toolkit (GATK)[8] was used for SNP calling. The Weir and Cockerham estimator of *F*_ST_ was calculated on the LW and MIN founder pigs on 100-kb windows[9]. The hybrid effects were estimated between LW and MIN 100-kb windows with varying level of genomic differentiation. The 100-kb windows were binned into 20 quantiles with increasing levels of *F*_ST_, and the λ parameter for the 135 traits was estimated on each *F*_ST_ bin. For each *F*_ST_ bin, the sex-specific λ was estimated on a subset of 100-kb windows that were classified into that *F*_ST_ bin (**Figure 2A**). The λ of the total population in an *F*_ST_ bin (**Figure 1B**) was calculated as a mean of male and female λ values in that *F*_ST_ bin. In the analysis, the λ of a 100-kb window was provided as the λ of an *F*_ST_ bin in which the window was grouped. The hybrid vigor effect was identified as λ > 33.5 in the total population. The hybrid depression effect was identified as λ < 33.5 and *F*_ST_ > 0.26, and the inbreeding depression effect was identified as λ < 33.5 and *F*_ST_ < 0.095. We further conducted a leave-10%-out analysis by randomly sampling 90% of the traits for the λ estimation (1,000 replicates).

**Recombination rate analysis**

The recombination rates in 100-kb windows were calculated as the ratio of the total number of recombination events in the paternal and maternal genomes to the total number (578 * 2) of paternal/maternal genome transmissions to the F2. The mean recombination rate was calculated on each of the twenty *F*_ST_ bins used in the above analysis.

**Female heterozygote deficiency analysis**

To compare the heterozygote frequencies of genomic SNPs in males and females, the F2 genomes were resequenced using the HiSeq2000 platform with libraries constructed with 500-bp inserts. We had applied a strict quality control on the SNP dataset before comparing the heterozygote frequency in males and females. First, we have developed a C++ program (filter_bam.cpp) to filter the BAM files to retain “uniquely mapped” read pairs, with a mapping quality >=30. The maximum insert size of read pairs and a maximum of SNP number in each read pairs were also considered. This step is very important because repetitive elements/large gene families/gene duplications/structural variations may introduce some mapping errors, causing false positives of heterozygotes in all samples. Second, the DNA sequence from the centromeric region of the Y chromosome share some sequence identities with those of other autosomes will cause male bias in the heterozygote frequency comparison, for example the chr1:92-93Mb region we identified in the excluding SNP list. These regions would contribute false positives of male heterozygote frequency increase if they were not excluded. These SNPs were provided in the file “excluded.snps.list” in the GitHub repository. Third, for a given SNP, we include a sample only when this genomic site was sequenced with a sequencing depth of 4-15× for that sample. Too low sequencing depth will lead to a low level of detection of heterozygotes, and excessively high sequencing depth may be caused by mapping of paralogous sequences unidentified in the first two steps. The genomic site of an individual passed this filtration was classified as a valid genomic site for that sample. Fourth, for each 100-kb window, the numbers of heterozygotes and homozygotes were tallied for each SNP and then aggregated to compute the heterozygote ratio (HR) for each sex. HR is defined as *heterozygotes*/(*heterozygotes* + *homozygotes*), where the *heterozygotes* and *homozygotes* represent the total number of valid heterozygotes and valid homozygotes in each 100-kb window.

The female heterozygote deficiency was calculated separately for rare and common SNPs using the following formula:

$$female heterozygote deficiency=\frac{\left[ HR\left( male \right)-HR\left( female \right) \right]*2}{HR\left( male \right)+HR(female)}$$

The source code for calculating the female heterozygote deficiency is available at https://github.com/xiehb-evolution/hybrid-effects. The parameters were supplied with “windowsize=100000 mindepth=4 maxdepth=15 F2male.txt F2female.txt” for the xie_unphased_vcf_for_heterozygote_stat program, where the F2males.txt and F2females.txt provide the ID lists for F2 males and females. The columns 5 to 11 (the number of segregating sites, the numbers of male homozygotes, male heterozygotes, female homozygotes and female heterozygotes, and the ratio of male heterozygotes and the ratio of female heterozygotes) in the output are used for the rare SNPs (MAF < 0.05) analysis. For common SNPs (MAF>0.05), the data are provided in the same format (7 columns per MAF bin), with each bin representing an incremental step of 0.05 in MAF.

**Heterozygote advantage analysis**

The relative fitness of heterozygotes (MIN/LW and LW/MIN) and homozygotes (MIN/MIN and LW/LW) was assessed by comparing their genotype frequencies in the F2 generation. Heterozygote advantage was defined as a scenario where the frequency of heterozygotes exceeded the frequencies of both homozygotes, while heterozygote disadvantage was characterized by a lower frequency of heterozygotes compared to both homozygotes (**Figure 3A**). F2 samples containing recombination events in either paternal or maternal genomes of a 100-kb window were removed during the analysis on the window. In the “All” group, the genotype frequencies were calculated in the total F2 population. For the “Male” and “Female” groups, the genotype frequencies were counted in the F2 subpopulation of the sex. Other patterns of genotype frequency differences (for example, P1 > P0 but P1 < P3) were excluded from this analysis. To evaluate the role of selection in shaping heterozygote dynamics, the heterozygote advantage/disadvantage ratio was computed across different *F*_ST_ bins to indicate the selective pressures acting on heterozygotes in each sex.

**Mid-parent heterosis and homozygote phenotypic difference analysis**

To explore the relationship between the distinct evolutionary hybrid effects identified in this study and the quantitative mid-parent heterosis (MPH), we analyzed the pattern of MPH of 135 traits across different *F*_ST_ bins. Since the F0 traits could not be measured under identical conditions due to differences in age and reproductive body condition, the mid-parent point in MPH was practically not available and thus was approximated using the mean phenotypic values of the F2 LW/LW and MIN/MIN homozygotes. A new statistic, the MPH’, was developed as the percentage deviation of the mean heterozygote phenotype from the mean homozygote phenotype, using the following formula:

$$MPH'=\frac{| Het-\frac{\left（ Homo1+Homo2 \right）}{2} |}{\left（ Homo1+Homo2 \right）/2}\times100\%$$

Here, the *Het* is the phenotypic mean for a heterozygous (LW/MIN or MIN/LW) genotype at a 100-kb window, and the *Homo1* represents the phenotypic mean for the LW/LW genotype and *Homo2* represents the phenotypic mean for the MIN/MIN genotype.

The phenotypic difference between homozygotes (MIN/MIN and LW/LW) was computed for each 100-kb window. To standardize comparisons across traits, this difference was normalized to a percentage by scaling it relative to the phenotypic mean within each sex.

**Data availability**

The Illumina Porcine SNP60 BeadChip data for the LW-MIN family are available at https://github.com/xiehb-evolution/hybrid-effects/blob/main/sscrofa11.data.zip. The whole genome resequencing data are deposited in the Genome Sequence Archive (GSA; http://gsa.big.ac.cn) under accession number CRA002451. The phenotypic data for the 135 traits are available at https://github.com/xiehb-evolution/hybrid-effects/blob/main/f2_trait_name_trait_value.csv.

**Code availability**

The core scripts for the hybrid effect analysis are available in a GitHub repository (https://github.com/xiehb-evolution/hybrid-effects).

**Supplementary Figures**

**
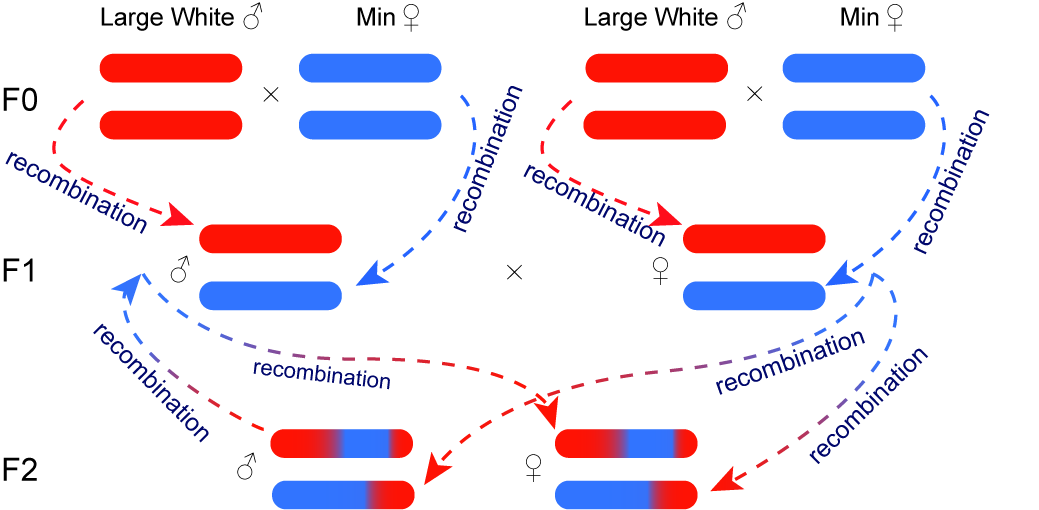
**

Figure S1. Schematic showing of the autosomal transmission in the LW-MIN family and determination of F2 genotypes in 100-kb sliding windows according to the allelic origin from the F0 founder breeds. Each individual was plotted with its paternal (above) and maternal (below) chromosomes. The genotype was determined as the paternal/maternal alleles with the F0 founder breed origin, with MIN/LW and LW/MIN being considered as different.


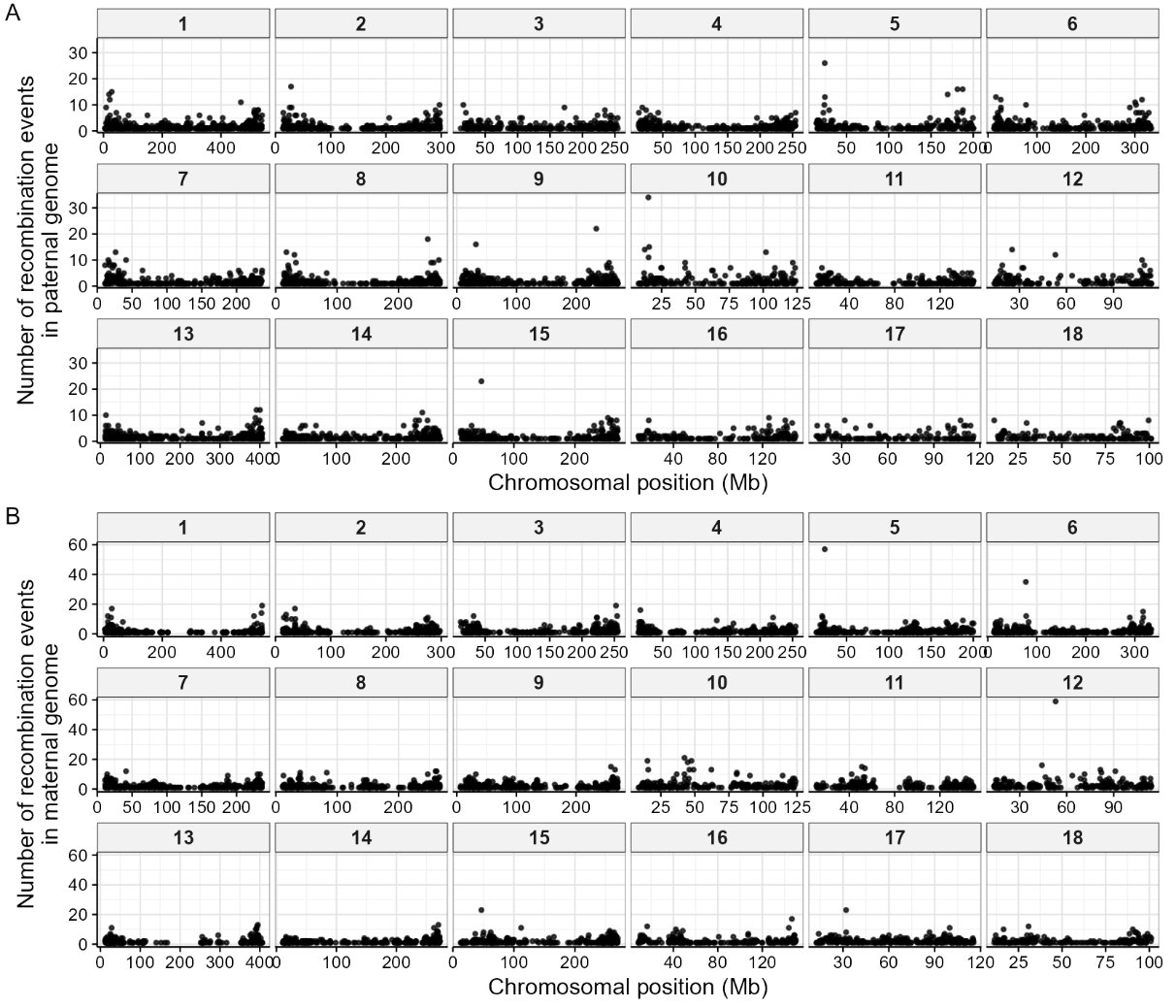


Figure S2. The recombination breakpoints inherited in the F2 paternal (A) and maternal (B) chromosomes were determined separately by resolving the F2 paternal or maternal haplotypes using the two haplotypes in the F1 males (paternal) and F1 females (maternal). The y-axis is the total number of recombination events identified in each of 100-kb sliding windows of the 578 F2 individuals. The data was plotted on the chromosomes 1 to 18. The recombination deserts on the maternal chromosomes 1 and 13 were shown in genomic region without data points (no recombination events).


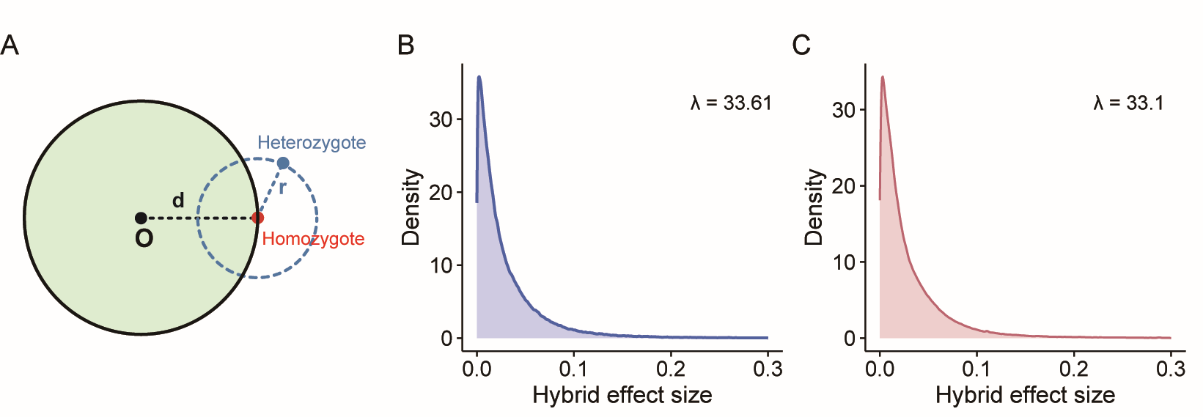


Figure S3. Fisher’s geometric model for hybrid effect analysis and the exponential distributions of hybrid effect size in 135 F2 traits. A. Fisher's geometric model used for hybrid effect analysis, treating the homozygote as the wild type and the heterozygote as a mutant type. *r* indicates the phenotypic difference (hybrid effect size) between a homozygote and a heterozygote in a 100-kb window. *O* and *d* indicate the phenotypic optimum of the model and the phenotypic distance of a homozygote to the optimum, respectively. The hybrid effect size distribution was analyzed separately for males (B) and females (C). The rate parameter (λ) was estimated based on hybrid effect sizes (≤ 0.3), which were scaled to the population mean for each sex.


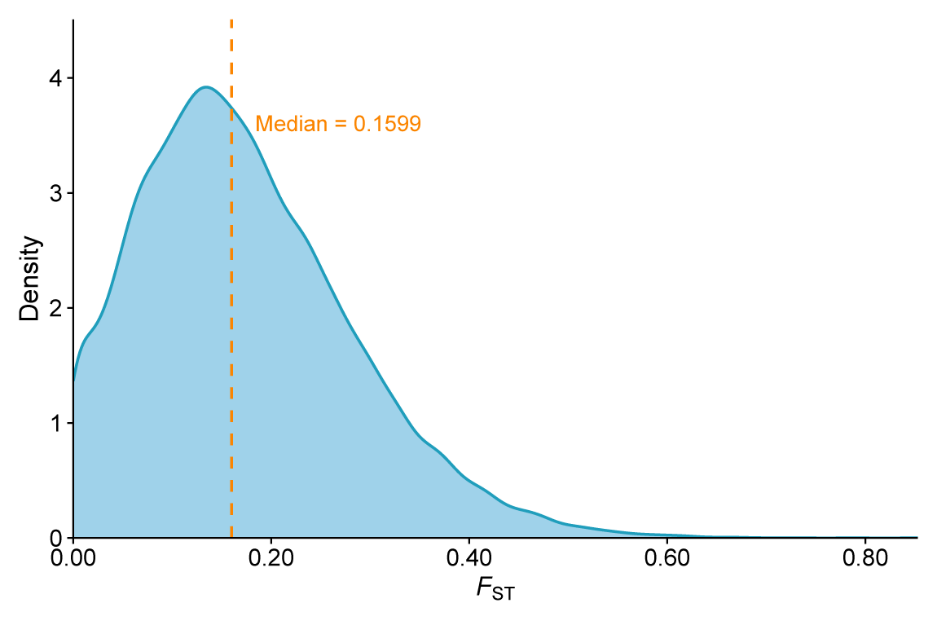


Figure S4. The distribution of *F*_ST_ between the LW and MIN founder pigs in 100-kb autosomal windows.


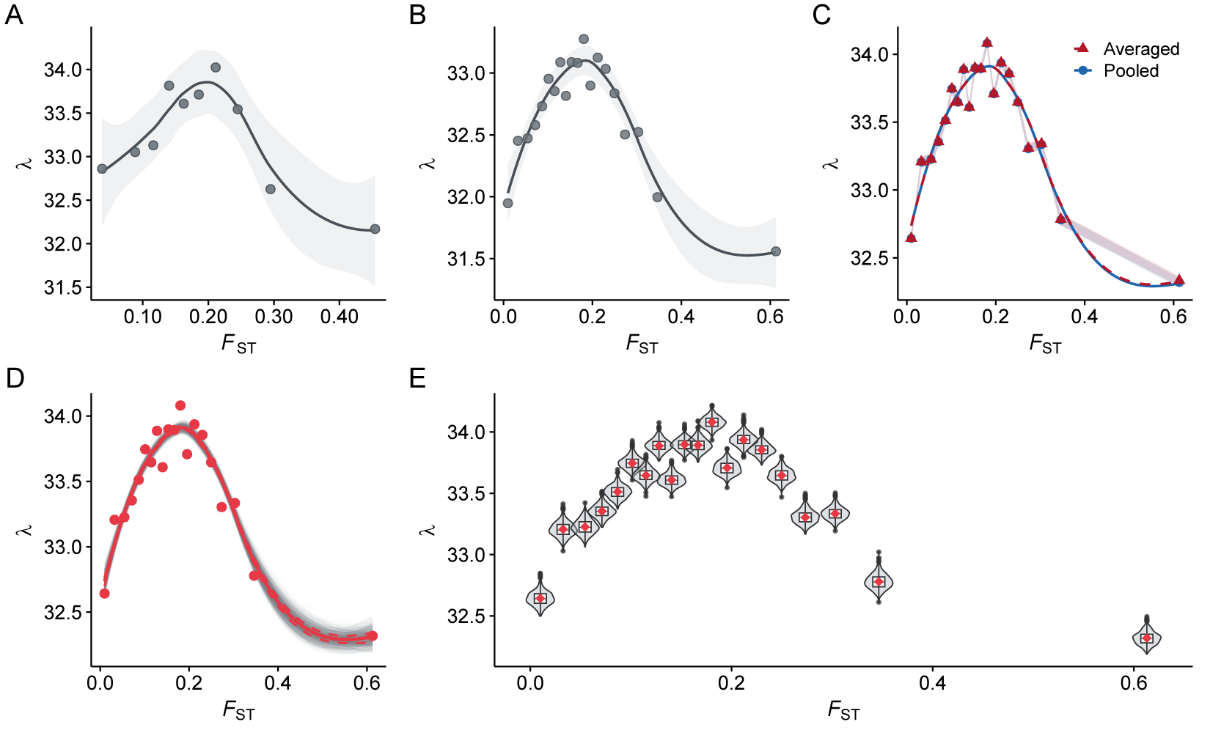


Figure S5. Validation of the hump-shaped distribution of λ across *F*_ST_ bins. A. Hump-shaped distribution of λ observed in 1-Mb sliding windows across ten *F*_ST_ bins. B. Hump-shaped distribution of λ in 100-kb windows using continuous traits only. C. Comparison of λ estimates using pooled versus averaged approaches across sexes. The averaged approach calculated sex-specific hybrid effect sizes, with λ for each bin estimated as the mean of λ values from males and females. D. Hump-shaped distribution of λ in 1,000 iterations of leave-10%-out analysis. In each iteration, 10% of traits were randomly excluded for λ estimation. E. Violin plot showing variation in estimated λ across *F*_ST_ bins in the leave-10%-out analysis.


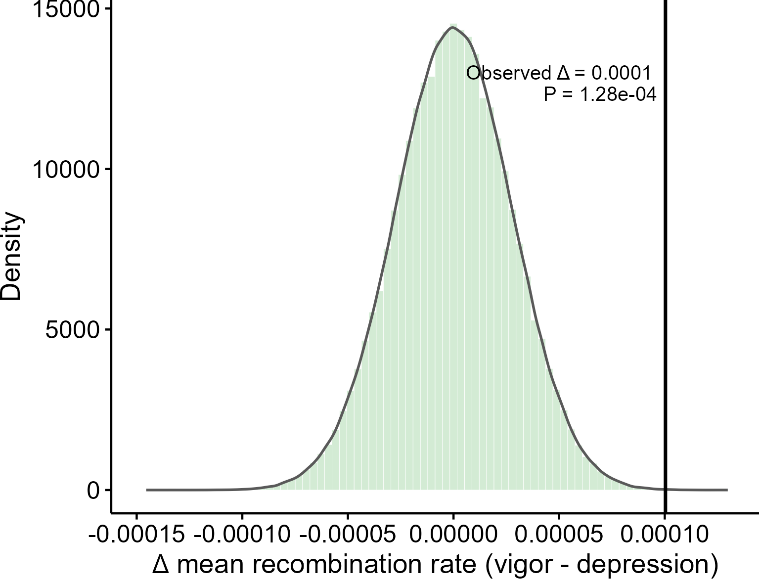


Figure S6. Permutation test for observing higher recombination rates in hybrid vigor regions than in inbreeding/hybrid depression regions. The permutation test was conducted with 1.0 × 10^6^ replicates. The *x*-axis indicates the recombination rate difference (Δ) between hybrid vigor and inbreeding/hybrid depression regions. The green vertical line indicates the observed difference.


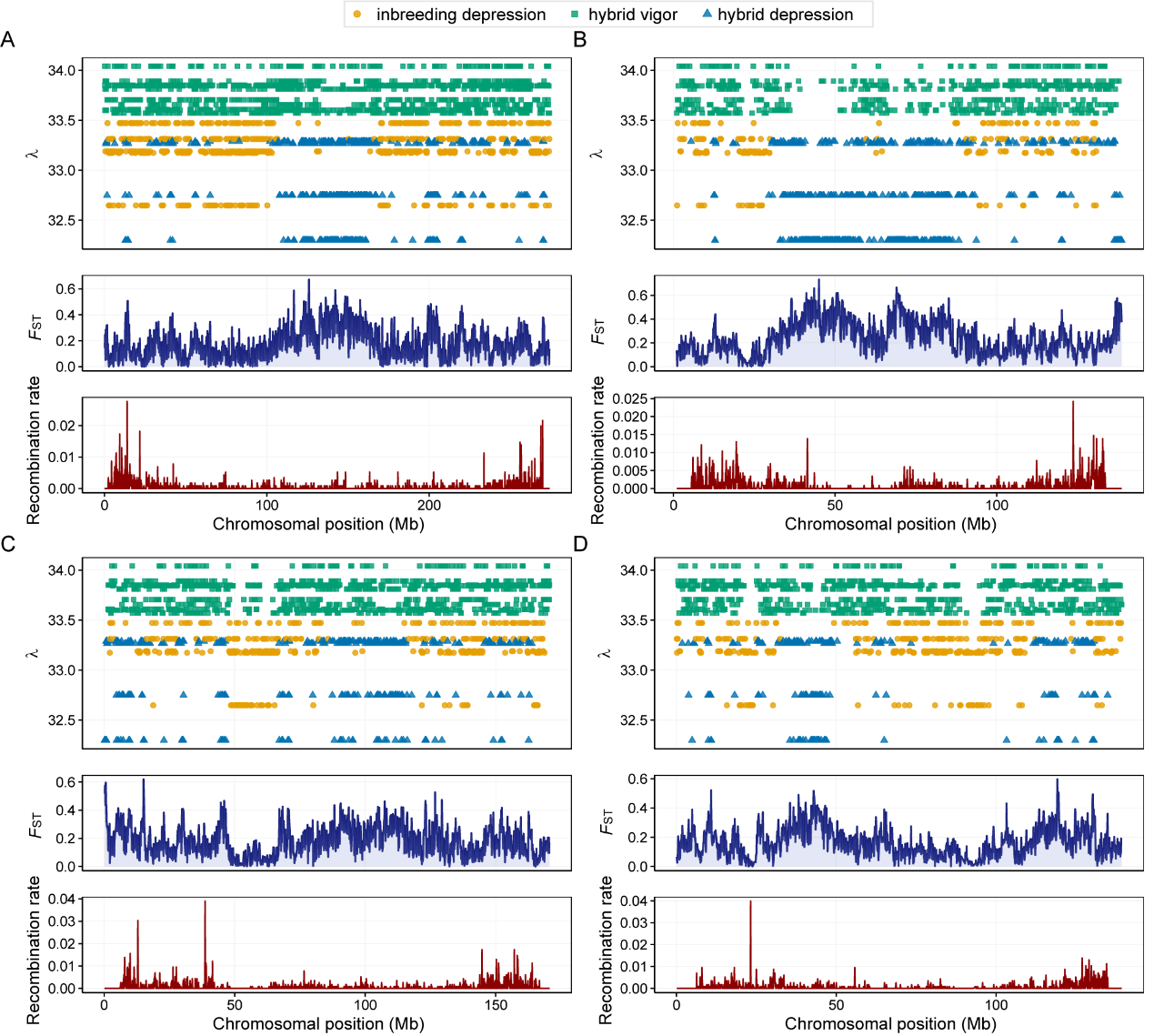


Figure S7. The hotspots of hybrid depression and inbreeding depression under low recombination rate with different levels of genomic differentiation. The hybrid depression effects (dark blue λ valley) are plotted on chromosomes 1 (A) and 8 (B), and the inbreeding depression (yellow λ valley) are plotted on chromosomes 6 (C) and 15 (D). The hybrid depression is characterized by low λ in long genomic regions with low recombination rates and high LW-MIN *F*_ST_. The inbreeding depression shows low λ in much shorter genomic regions with low level of *F*_ST_.


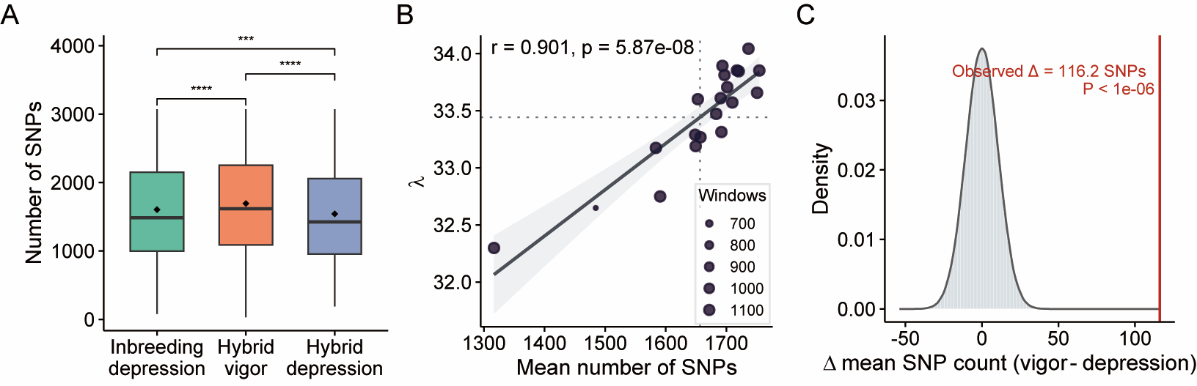


Figure S8. Heterogeneous evolutionary rates under the hybrid effect variation. A. The hybrid vigor region (selected with λ > 33.5) exhibits a significantly higher SNP density in 100-kb windows compared to the inbreeding depression and hybrid depression regions. B. The recombination-mediated hybrid effect variation drives heterogeneous genome evolution, as evidenced by a positive correlation between SNP density and the λ parameter. C. Permutation test for observing 116.2 more SNPs in hybrid vigor regions than in inbreeding/hybrid depression regions. The *x*-axis indicates the difference in SNP number (Δ) between hybrid vigor and inbreeding/hybrid depression regions. The vertical red line indicates the observed SNP number difference.


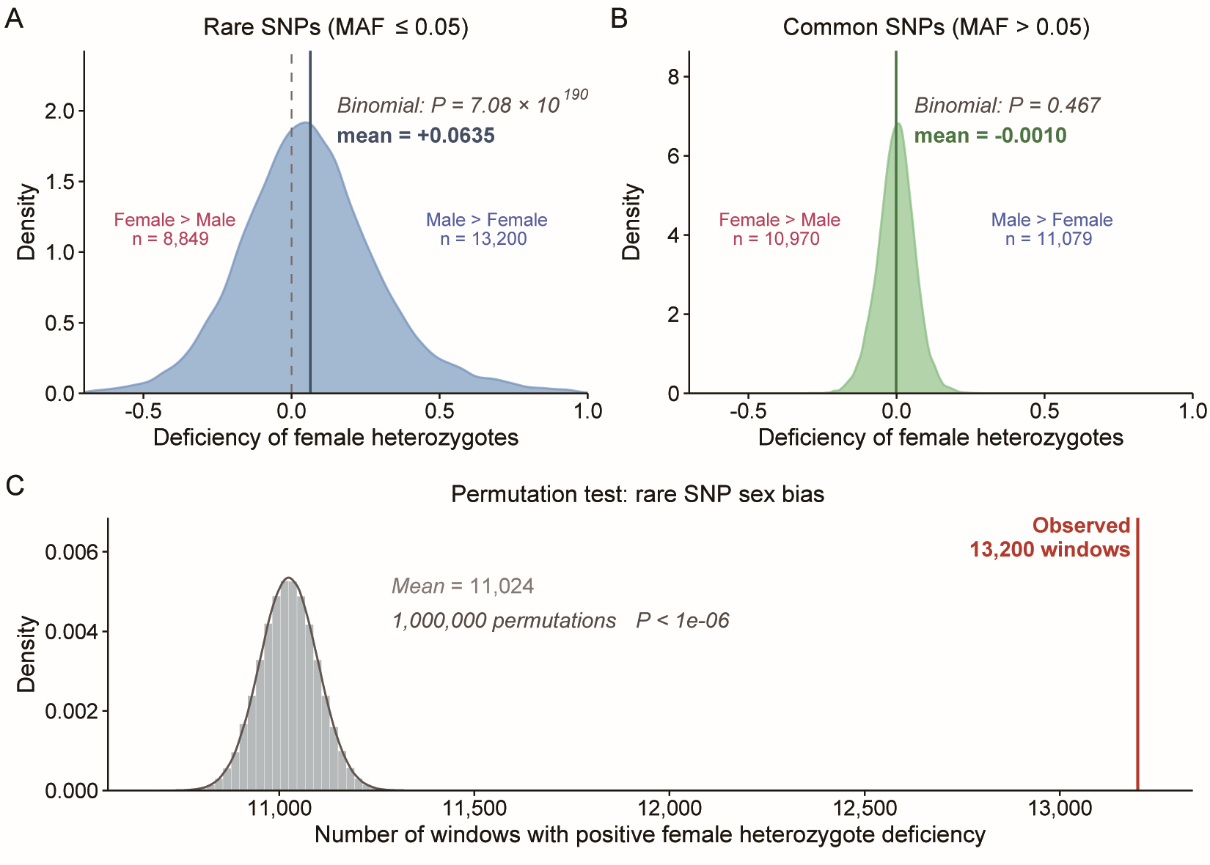


Figure S9. Deficiency of female heterozygotes summarized from whole-genome resequencing data. A. Density plot of 100-kb windows across different levels of female heterozygote deficiency for rare SNPs (MAF ≤ 0.05). Female heterozygote deficiency was calculated as the sex difference in heterozygote ratio (HR) in the F2 population using the formula [HR(male) – HR(female)] / [(HR(male) + HR(female))/2]. B. Density plot of 100-kb windows across different levels of female heterozygote deficiency for common SNPs (MAF > 0.05). C. Permutation test for observing 13,200 and 8,849 windows with positive and negative female heterozygote deficiency values, respectively.


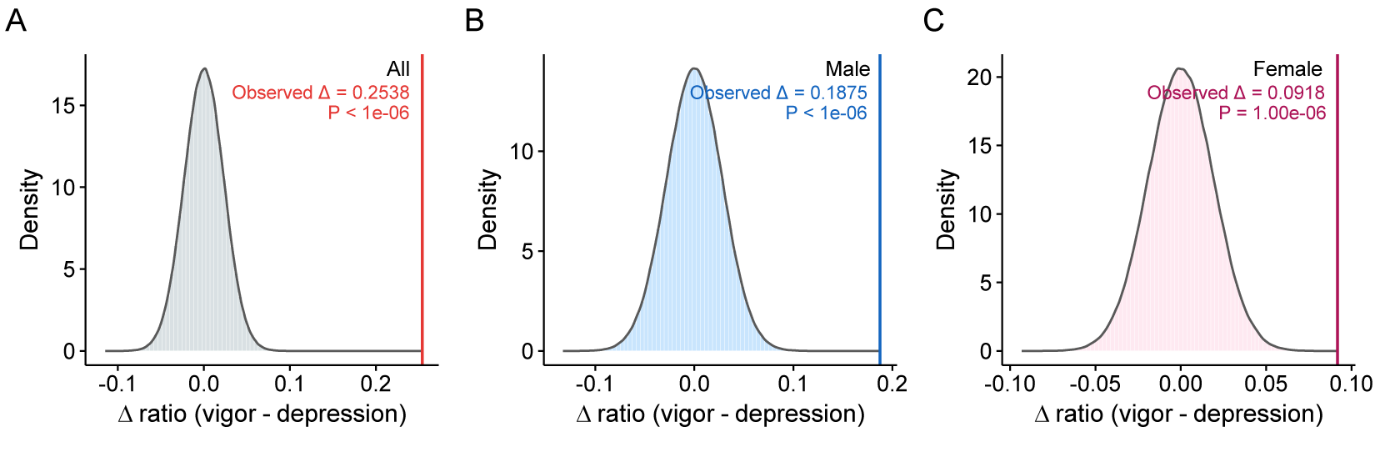


Figure S10. Permutation test of observing an increased heterozygote advantage/disadvantage ratio in hybrid vigor regions compared with inbreeding/hybrid depression regions. The permutation test was conducted with 1.0 × 10^6^ replicates for the total population (A), males (B) and females (C). The x-axis indicates the heterozygote advantage/disadvantage ratio difference (Δ) between hybrid vigor and inbreeding/hybrid depression regions. Vertical lines indicate the observed differences.

**Supplementary Tables**

Table S1. Distribution of recombination breakpoints in the F2 genomes during the genomic transmission from F1 to F2 (see Excel file ‘supplementary data.xlsx’).

Table S2. A summary of 135 traits collected in the F2 population from the LW-MIN family (see Excel file ‘supplementary data.xlsx’).

Table S3. The distribution of autosomal 100-kb windows in the three categories of hybrid effects (see Excel file ‘supplementary data.xlsx’).

Table S3. The number of windows and traits used for *F*_ST_ bin analysis (see Excel file ‘supplementary data.xlsx’).

Table S4. The distribution of autosomal 100-kb windows in the three categories of hybrid effects (see Excel file ‘supplementary data.xlsx’).

Table S5. The father-to-son and mother-to-daughter transmission of recombination events in 157 windows with *F*_ST_ > 0.5. (see Excel file ‘supplementary data.xlsx’).

Table S6. The distribution of heterozygote advantage and disadvantage windows across the *F*_ST_ bins. (see Excel file ‘supplementary data.xlsx’).

**References**

1. O'Connell J, Gurdasani D, Delaneau O *et al.* A general approach for haplotype phasing across the full spectrum of relatedness. *PLoS Genet*. 2014; **10**(4): e1004234. doi: 10.1371/journal.pgen.1004234

2. Fisher RA. *The genetical theory of natural selection*. Oxford: Oxford University Press, 1930.

3. Delignette-Muller, Marie Laure, Dutang C. fitdistrplus: An R package for fitting distributions. *Journal of statistical software*. 2015; **64**: 1-34.

4. Orr HA. The population genetics of adaptation: the distribution of factors fixed during adaptive evolution. *Evolution*. 1998; **52**(4): 935-949. doi: 10.1111/j.1558-5646.1998.tb01823.x

5. Groenen MA, Archibald AL, Uenishi H *et al.* Analyses of pig genomes provide insight into porcine demography and evolution. *Nature*. 2012; **491**(7424): 393-398. doi: 10.1038/nature11622

6. Li H, Durbin R. Fast and accurate short read alignment with Burrows-Wheeler transform. *Bioinformatics*. 2009; **25**(14): 1754-1760. doi: 10.1093/bioinformatics/btp324

btp324 [pii]

7. Li H, Handsaker B, Wysoker A *et al.* The Sequence Alignment/Map format and SAMtools. *Bioinformatics*. 2009; **25**(16): 2078-2079. doi: 10.1093/bioinformatics/btp352

8. McKenna A, Hanna M, Banks E *et al.* The Genome Analysis Toolkit: a MapReduce framework for analyzing next-generation DNA sequencing data. *Genome Res*. 2010; **20**(9): 1297-1303. doi: 10.1101/gr.107524.110

9. Danecek P, Auton A, Abecasis G *et al.* The variant call format and VCFtools. *Bioinformatics*. 2011; **27**(15): 2156-2158. doi: 10.1093/bioinformatics/btr330
